# Bempedoic acid: A nonstatin drug for the management of hypercholesterolemia

**DOI:** 10.1002/hsr2.431

**Published:** 2021-11-09

**Authors:** Asvitha Govindaraju, Sarvesh Sabarathinam

**Affiliations:** ^1^ Department of Pharmacy Practice SRM College of Pharmacy, SRM IST Kancheepuram India

## 1 INTRODUCTION

Statins, or β‐hydroxy β‐methylglutaryl‐CoA (HMG‐CoA) reductase inhibitors, are considered as the first‐line treatment for the management of dyslipidemia in terms of reduction in the low‐density lipoprotein cholesterol (LDL‐C) level and impeding the cardiovascular events.^1^ In many countries, statins are the most recommended medication; numerous clinical trials have consistently proven that statins could play a remarkable role in the reduction of cardiovascular events.^2^ Statins are usually effective and well‐tolerated, although they are not appropriate for all individuals.^3^ Statins can induce rhabdomyolysis, hepatic dysfunction, renal failure, and other side effects. Statin intolerance has been reported in about 5% and 20% of individuals, which further lead to statin discontinuation.^4^ To attain the significant changes in individuals with statin resistance, an alternative nonstatin should be practiced (bempedoic acid). This review discusses the overview of bempedoic acid.

### 1.1 Overview of Bempedoic acid

Bempedoic acid (BA) was approved by the USFDA in the year 2020 as a nonstatin for hypercholesterolemia. It is an oral agent that inhibits adenosine triphosphate citrate lyase, which is an enzyme involved in cholesterol synthesis by catalyzing acetyl‐CoA.^5^ BA is activated by the enzyme acyl‐CoA synthetase, which in humans is encoded by the gene *SLC27A2* and primarily expressed in the liver and kidney.^6^ Molecular weight of BA was found to be 344.492; logP is 4.4699 with 14 rotatable bonds along with 3 acceptors and 3 donors. The water solubility of BA is −2.935 (log mol/L), Caco_2_ permeability was 0.591 (log Papp in 10^−6^ cm/s), skin permeability is −2.735 (log kp), BA is not a substrate of P‐glycoprotein, and BA is not a inhibitor of P‐glycoprotein ‐I and II. While coming to distribution fraction unbound in human was 0.273 (Fu), blood–brain permeability was −1.205 (log BB), and central nervous system permeability was −3.14 (log PS). BA is not a substrate and inhibitor of CYP2D6, CYP3A4, CYP1A2, CYP2C19, CYP2D6, and CYP3A4 isoenzymes. The maximum tolerated dose in human is −0.498 (log mg/kg/d), Acute fathead minnow toxicity was −0.722 (log mM) and BA is not an inhibitor of hERG I and II inhibitors.^7^

The activated substance inhibits adenosine triphosphate citrate lyase, which is involved in the liver's biosynthesis of cholesterol upstream HMG‐CoA reductase, the enzyme that is inhibited by statins. The half‐life of BA is 15‐24 hours, and the site of absorption is the small intestine. Bempedoic acid has been shown to provide a gradual decrease in LDL‐C when used in fusion with both statins and ezetimibe at all doses.^8^ Though bempedoic acid acts on the same cholesterol biosynthesis pathway as statins, the muscle‐related adverse event rate in CLEAR Serenity with bempedoic acid was not activated in skeletal muscle and did not differ from placebo, even among patients who had experienced muscle‐related symptoms while on statin therapy.^9^

### 1.2 Clinical evidence of bempedoic acid

The initial dose of bempedoic acid is 180 mg/d. BA is a small molecule that is to be taken once daily orally that is absorbed through the small intestine. Once taken up by the liver, the half‐life varies between 15 and 24 hours. Initial studies reported up to a 27% decrease in LDL‐C with BA used as monotherapy, up to 24% additional decrease in LDL‐C when used with statins, and up to 48% total decrease with ezetimibe.^10^, ^11^ Existing reports say that use of bempedoic acid may increases the uric acid and creatinine.^12^

### 1.3 Increase uric acid leads to

1. Endothelial dysfunction: Uric acid nurtures hypertension due to increased systemic vascular resistance also by reducing the bioavailability of nitric oxide.
2. Inhibiting insulin pathway: Uric acid inhibits the trigger of an insulin signaling pathway.
3. Thrombus platelet aggression.
4. Uric acid contributes activation of RAAS by increasing juxtaglomerular renin. In diabetes, RAAS can cause, vascular dysfunction and inflammation leading to renal AND cardiovascular complications.^13^, ^14^

## 2 DISCUSSION

This review includes patients with a high risk of cardiovascular diseases, patients already on statin therapy, patients who take ezetimibe. Many studies proved BA as an essential drug of choice for patients who are intolerant to statins (Table 1). Some systematic reviews conclude about the effects of BA. Ray et al concluded that the use of BA increases uric acid and GOUT but also said that it is reversible after discontinuation.^18^, ^19^, ^20^ Arikeri and Punukula concludes that BA might be safe in combination with statins as well as ezetimibe and appears to be effectively lower LDLC and has the potential to reduce the risk of muscle‐related adverse events.^15^ Ballantyne et al concludes that BA+EZE provides a potent therapy that is complementary to existing lipid‐modifying regimens.^16^ Goldberg et al concludes that patients at higher risk for CVD receiving maximally tolerated statins, along with BA compared with placebo resulted in a significant lowering of LDL‐C level over 12 weeks.^17^ There were no new oral nonstatin options for LDL‐C lowering in nearly 20 years, and Nexletol (BA) is an anticipated solution for the appropriate patients that have not been able to reach their LDL‐C goals and could benefit from Nexletol immediately. The drug's approval by the US Food and Drug Administration comes after studies showed an 18% to 28% fall in LDL cholesterol, compared with placebo, in patients who were also on statin. BA cost is approximately 374 USD for 30 tablets. Even though BA might have effects in increasing UA and creatinine, but it is highly reversible and is safe using BA as monotherapy or in combination with atorvastatin or ezetimibe. Using BA in combination especially with atorvastatin helps decrease LDL‐C faster and also avoid other muscle‐related adverse events.

**TABLE 1:** Clinical evidences of bempedoic acid

S.no	Reference	Dose	Study design	Duration	Parameter	Outcome
1.	Arikeri and Punukula	BA‐180 mg (n = 45) + ATV‐80 mg (n = 23)	Open labeled, randomized	4 wk	LDL‐C	Significant reduction in LDL‐C levels.
2.	Ballantyne et al^16^	BA‐180 mg (n = 110) + EZE‐10 mg (n = 109)	Multicenter, double‐blind study	12 wk	LDL‐C	BA+EZE lowered LDL‐C levels.
3.	Goldberg et al^17^	BA‐180 mg (n = 522) Placebo (n = 257)	Randomized, double‐blind, placebo‐controlled clinical trial	12 wk	LDL‐C	Addition of BA decreased LDL‐C levels.

Abbreviations: BA, bempedoic acid; LDL‐C, low‐density lipoprotein cholesterol.

## 3 CONCLUSION

Statin is a golden standard drug, for the management of hyperlipidemia, which has a dual role in the reduction of bad cholesterol and increase in HDL. However, 50% of patients are intolerant to statins because of their frequently reported ADR's in a higher ratio. The long‐term safety and efficacy of BA, including effects on cardiovascular outcomes, are currently being assessed in phase 3 clinical trials. This outcome study will be important for determining the function of BA as a suitable alternative for statin intolerance.

## FUNDING

This research received no grant from any funding agency.

## CONFLICT OF INTEREST

The authors declare no conflict of interest, financial, or otherwise.

## AUTHOR CONTRIBUTIONS

Conceptualization: Sarvesh Sabarathinam.

Supervision: Sarvesh Sabarathinam.

Writing and editing: Asvitha G.

All authors have read and approved the final version of the manuscript.

## TRANSPARENCY STATEMENT

The corresponding author confirms that the manuscript is an honest, accurate, and transparent account of the study being reported.
